# Experimental data on the degradation of caffeine by photo-electro-fenton using BDD electrodes at pilot plant

**DOI:** 10.1016/j.dib.2018.10.174

**Published:** 2018-11-03

**Authors:** Natalia Lopez-Saavedra, Luis F. Muñoz-Delgado, Jose A. Lara-Ramos, Fiderman Machuca-Martinez

**Affiliations:** Escuela de Ingeniería Química, Universidad del Valle, A.A. 23360 Cali, Colombia

## Abstract

Emerging contaminants (EC) are an imminent risk due to potential toxicity to aquatic ecosystems and human beings. This type of contaminants is found in low concentrations and usually present incomplete or inefficient removal by conventional treatments, which entail its permanence and constant increase. Advanced Oxidation Processes (AOP) are an alternative for the elimination of dangerous and resistant substances in wastewater. So, this research evaluates the caffeine degradation in aqueous solution by AOP, such as: Fenton, Electro-Oxidation (EO) with boron doped diamond (BDD) electrodes, Electro-Fenton (EF) and Photo-Electro-Fenton (PEF). The influences of pH, concentration of the supporting electrolyte and specific electric charge were investigated using a Taguchi׳s factorial design, which allowed to identify the contribution of each variable in the process. The data obtained in this work can be useful for scaling process and cost analysis because it provide the information at pilot plant scale.

**Specifications table**

**Table:** 

Subject area	Chemical engineering
More specific subject are	Advanced oxidation process
Type of data	Figure and table
How data was acquired	Data were obtained by Cyclic voltammetry, differential pulse, UV–vis spectrophotometry, total organic carbon (TOC) and chemical oxygen demand (COD).
Data format	Analyzed
Experimental factors	All experimental tests were performance to pilot scale in a reactor equipped with a cell of six BDD electrodes (three anodes and three cathode). For Photolysis, Photo-Fenton and PEF tests were used a UV lamp (20 W). Influences of pH, concentration of the supporting electrolyte and specific electric charge were investigated. A factorial design Taguchi were used for obtained experimental data.
Experimental features	Caffeine degradation by different AOP were investigated.
Data source location	GAOX, Universidad del Valle, Cali, Colombia.
Data accessibility	The data is found only in this article.

**Value of data**

• Taguchi design was a useful statistical method to find the best operating conditions in the degradation of caffeine.
• The potentials of oxidation and reduction of the species in solution allows to know the voltage suitable for carried out the test and save time and money.
• Data obtained show that PEF process is better method to the caffeine degrade in aqueous solution.
• Data may be useful for scaling process and cost analysis.

## 1 Data

**Fig. 1:**
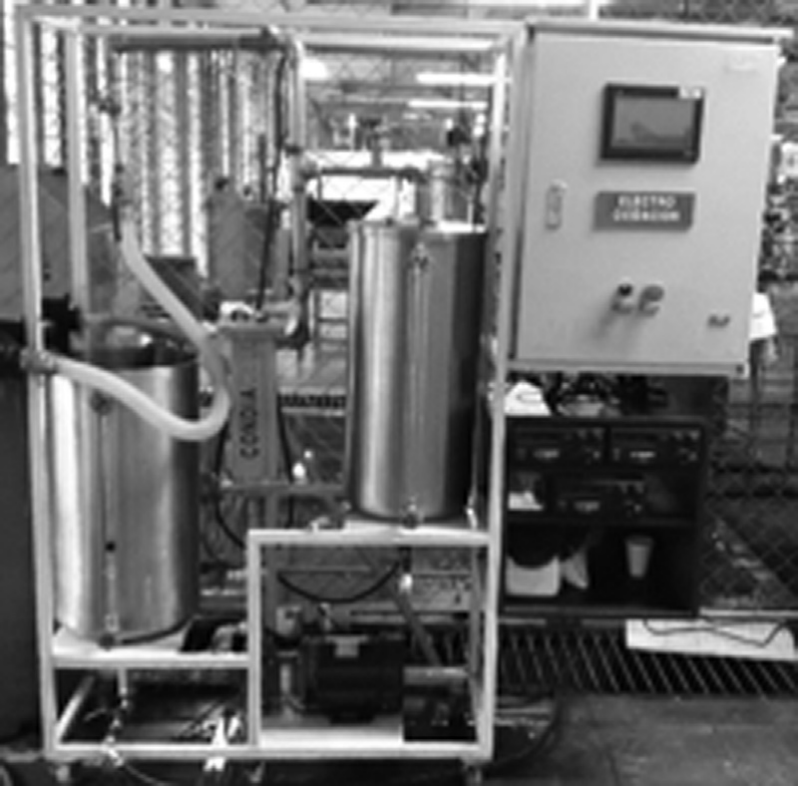
Photography of the pilot plant.

This brief data set describes the use of the PEF process for caffeine degradation present in water. Table 1 shows the physical and chemical properties of caffeine, Table 2 shows the experimental factors and assigned values in each level, Table 3 contains the statistical design matrix and the Fig. 1 presents a photography of the reactor.

**Table 1:** Physicochemical properties of caffeine [1], [2].

Molecular structure	
Molecular formula	C_8_H_10_N_4_O_2_
Molecular weight (g mol^−1^)	194.194
Melting point (°C)	236.2
Water solubility (mg L^−1^ at 25 °C)	21600
pKa (at 25 °C)	14.0

**Table 2:** Factors and levels of the experimental design [1], [2].

Factors	Levels	
	Low	High
pH	2.8	3
Concentration of supporting electrolyte (mM)	17	20
Specific electrical load (A h L^−1^)	2	3

**Table 3:** Taguchi design matrix.

Specific electrical charge (A h L^−1^)	pH	Concentration of supporting electrolyte (Na_2_SO_4_ in mM)
2	2.8	17
2	2.8	17
2	2.8	20
2	3	20
2	3	17
2	3	20
3	2.8	20
3	2.8	17
3	2.8	20
3	3	17
3	3	20
3	3	17

**Fig. 2:**
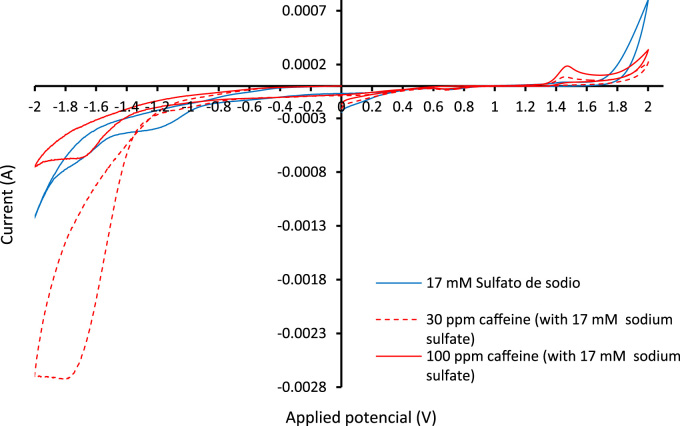
Cyclic voltammogram of caffeine at two concentrations and of the electrolyte support with a scanning speed was 100 mV s^−1^ and pH = 3.

**Fig. 3:**
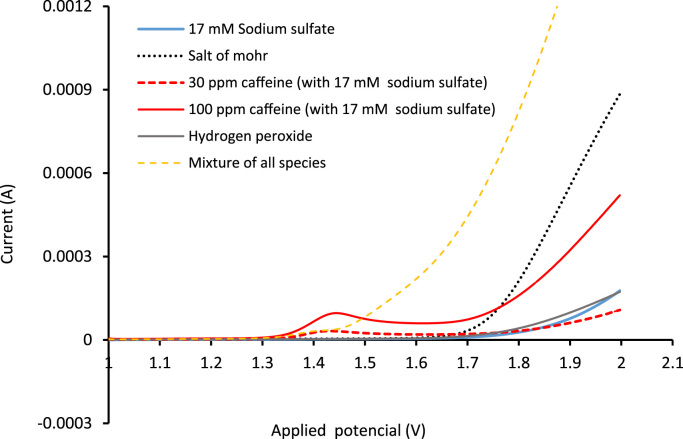
Anodic differential pulse technique for each of the chemical species in solution and of the mixture with a scanning speed of 100 mV s^−1^ and pH = 3.

**Fig. 4:**
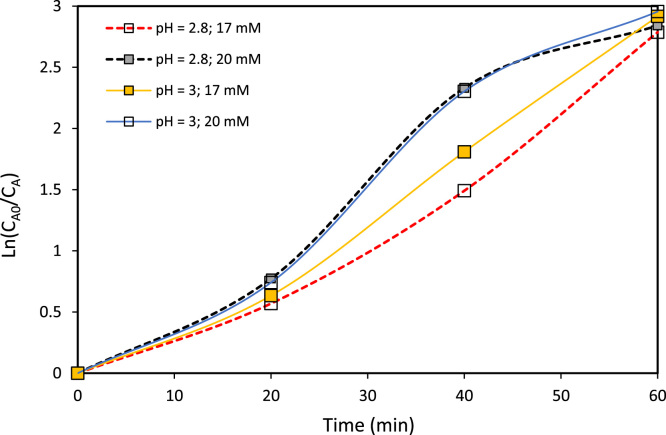
Degradation of caffeine (*C*_A0_ = 30 mg L^−1^) by PEF process with specific electric charge Q = 2 (A h L^−1^) at different pH values and concentration of electrolytic support.

**Fig. 5:**
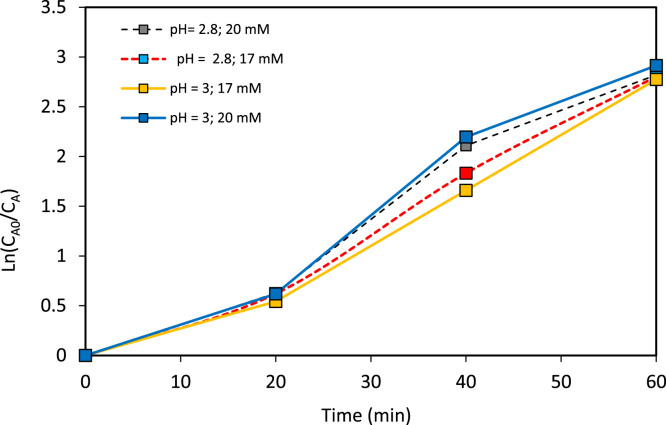
Degradation of caffeine (*C*_A0_ = 30 mg L^−1^) by PEF process with specific electric charge *Q* = 3 (A h L^−1^) at different pH values and concentration of electrolytic support.

**Fig. 6:**
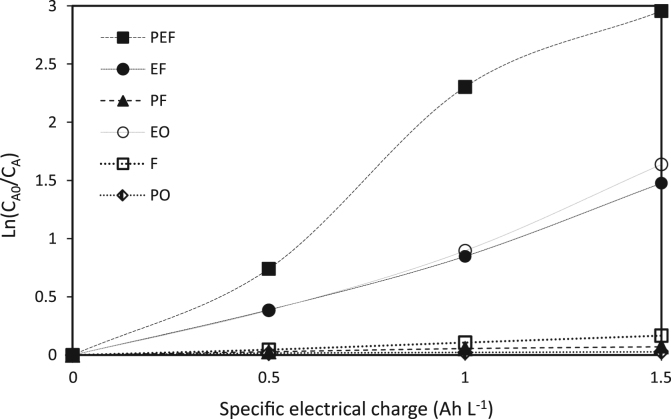
Degradation of caffeine by Photolysis (PO) and oxidative methods: electro-oxidation (EO) and Fenton (F), and combined Photo-Fenton (PF), Electro-Fenton (EF) and Photo-Electro-Fenton (PEF).

The verification of the anodic oxidation potential (cyclic voltammogram) of caffeine and of the supporting electrolyte is illustrated in Fig. 2, the behavior of the supporting electrolyte, caffeine, salt Mohr, hydrogen peroxide and the mixing of all when the anodic differential pulse technique is applied is shown in Fig. 3, the variation of the caffeine concentration for specific electric charge of 2 and 3 A h L^−1^ can be seen in Fig. 4, Fig. 5 respectively; alike, the profile of caffeine degradation by each oxidative process are show in Fig. 6. Also, the variation of the total organic carbon (TOC) and the chemical oxygen demand (COD) under the best conditions are included in Table 4.

**Table 4:** Conditions that present better degradation with specific electric charge *Q* = 2.

TOC_Initial_ (ppm)	COD_Initial_ (ppm)	pH		[Na_2_SO_4_] (mM)	% TOC	% COD
18.45	21.07	3.0		17	38.43	38.46
18.17	25.66	3.0		20	56.01	77.27
Conditions that have better degradation with specific electric charge *Q* = 3.						
15.29	22.69		2.8	17	64.72	64.29
14.70	21.07		3.0	17	71.41	76.92

## 2 Experimental design, materials and methods

For the development of the experimental section of this work was used the Taguchi factorial design [3]. This design uses an orthogonal arrangement which recognizes that not all the causative factors of variability can be controlled [1]. From this matrix the best design parameters for the description of the initial stage of the caffeine degradation by PEF were found.

A through analytical techniques such as cyclic voltammetry and differential pulse the oxidation potential (on BDD electrodes) for the chemical species present in solution (Sodium sulfate, Na_2_SO_4_, hydrogen peroxide, H_2_O_2_, Caffeine and Fe^+2^ contained in Mohr׳s salt) were corroborate with the reported literature. For these measurements, it was used the potentiostat Gamry® (galvanostat series G750).

Then tests were performed in reactor with BDD electrodes taking into account the minimum oxidation value of each species, and the production potential of hydroxyl radical. In addition, it was verified the synergy of the oxidative methods (PO, EO, Fenton, FF and PEF) with respect PEF.

On the other hand, the caffeine concentration was quantified using a UV–vis spectrophotometer (SHIMADZU®) at a wavelength of 273 nm, which was found with the spectral sweep. The test that showed the best degradation were subjected to two types of analysis more, chemical oxygen demand (COD), which we determined by the open reflux method [4] and mineralization through measurements of total organic carbon (TOC) in a TOC-V CPH SHIMADZU® analyzer [5].

Also, the generation of H_2_O_2_ was verified by UV–vis spectrophotometry at a wavelength of 408 nm. This measurement methodology is based on the photoelectric quantification of the color intensity of the H_2_O_2_ solutions treated with titanium sulfate reagent, which produced a yellow color in the reaction, this color is due to the formation of per titanic acid (H_2_TiO_4_). For this experimental process, 1 mL of sample and 1 mL of titanium sulfate reagent were added to 10 mL graduated balloon and completed with distilled water up to its capacity. After of 5 min, the measurement of the solution in the spectrophotometer was performed [6].
