# Supplementary material for: Experimental data on the degradation of caffeine by photo-electro-fenton using BDD electrodes at pilot plant
Source: Data Brief. 2018 Nov 3;21:1709–15. doi: 10.1016/j.dib.2018.10.174 (PMC6249512; doi:10.1016/j.dib.2018.10.174)
Supplement: Supplementary file 1 — Transparency document [file mmc1.docx]

Santiago de Cali, 29/09/2018

Data in Brief

# Editors

Dear Editors

The authors of the paper entitled: *EXPERIMENTAL DATA ON THE DEGRADATION OF CAFFEINE BY PHOTO-ELECTRO-FENTON USING AN BDD PILOT PLANT* by López Saavedra Natalia Muñoz, Delgado Luis Fernando, Jose Antonio Lara Ramos, and Fiderman Machuca Martínez do not have conflicts of interest for the publication of this work

Yours sincerely

Prof. Fiderman Machuca Martinez

Corresponding author
